# Digital economy, green finance, and economic resilience

**DOI:** 10.1371/journal.pone.0314028

**Published:** 2025-02-06

**Authors:** Jianqi Zhang

**Affiliations:** School of Economics, Yunnan Normal University, Kunming,Yunnan, China; Abu Dhabi University, UNITED ARAB EMIRATES

## Abstract

With the rapid development of digital technology, the digital economy has become an important force to promote economic growth and drive innovation, and to enhance economic quality and ecological efficiency through green finance. Additionally, green finance, as an important means to achieve resource and environmental sustainability, has received increasing attention and importance from the international community. This study explores how the digital economy and green finance contribute to economic resilience using panel data from 30 provinces and cities in China from to 2011–2023. The development of the digital economy can effectively promote economic resilience, and green finance plays a significant mediating role between the digital economy and economic resilience. In this regard, China’s economic resilience must be enhanced by strengthening the construction of digital infrastructure, promoting innovation and the development of green finance, and formulating a policy environment conducive to the development of green finance.

## 1. Introduction

With the dual impact of increasing uncertain risks in the global economy and downward pressure on China’s economy, long-term sustainable development requires that the economy remain resilient enough to withstand external risk shocks, while also combining the ability to recover quickly and restructure from shocks (Zhu Honghui et al., 2023) [1]. China’s current overall economic volume ranks second in the world, while the average annual growth rate of the economy is more than 5%, indicating that China’s economy is more resilient, which is also an important key for China to achieve high-quality development (Guan Changling et al., 2021) [2] and to achieve the optimization and upgrading of the country’s overall economic structure and transformation (Zhang Xuebo et al., 2023) [3]. However, considering that China’s economic space is characterized by its large scale, multi-levels, and heterogeneity (Xia Tim et al. 2024) [4], reducing development differences between Chinese regions and enhancing regional economic resilience is key to achieving China’s high-quality development.

Since its introduction, the connotation of digital economy has been continuously enriched and extended, which not only involves factors of production, digitalization of industries, digital industrialization and governance in generative relationships (Wang Yanchuan, 2024) [5], but also manifests itself in the deep integration and development of various industries in all fields of the economy. This effectively breaks down information barriers and perfects the efficiency of allocating factor resources, which has become an important way to optimize China’s economy under a “new normal”. It has become an important way to optimize industrial structure, integrate factor resources, and promote high-quality economic development under this new normal regarding China’s economy. Among the aspects of the digital economy in the financial field, green finance relies on big data and other digital technologies to identify green industries more accurately, innovate financial products, improve risk identification and assessment capabilities, and reduce information asymmetry between borrowers and lenders. It guides the precise flow of green financial products to green innovation and ESG enterprises, and breaks the financing constraints of easing to achieve an advanced and rationalized industrial structure. Therefore, an in-depth study of the digital economy’s impact on green finance and the intermediary role of green finance in realizing economic resilience is of great significance for narrowing the differences in China’s economic development and enhancing its overall economic resilience.

The existing literature on the digital economy, green finance, and economic resilience mainly focuses on impact studies between the two elements. Few studies have examined the digital economy’s impact on green finance. Chen et al. (2022) [6] find that the digital economy promotes the high-quality development of green finance by improving the information matching rate, integrating financial resources, and enhancing the universality of financial services. Qian et al. (2020) [7] argues that the digital economy promotes the development of green finance using its own digital tools. Tian et al. (2022) [8] and Wang et al. (2023) [9] argue that the digital economy can prompt consumers to effectively improve the allocation and utilization of resources and improve the efficiency of green financial resources in Chinese provinces. Regarding the impact of the digital economy on economic resilience, Deng Xiaole et al. (2024) [10] find, based on empirical tests, that the development of the digital economy can effectively promote China’s trade resilience. Yanchao et al. (2024) [11] find that the digital economy is an important driving force in enhancing the resilience of a city’s export-oriented economy. Tang’s (2023) [12] empirical test finds that the digital economy enhances the resilience of the tourism economy by stimulating tourism financial expenditure, guiding tourism industry clusters, increasing tourism consumption, and has positive spatial spillover effects (Zhu Jinhe et al., 2021) [13]. Wei et al. (2023) [14] argues that the impact of the digital economy on the resilience of the agricultural economy not only has a direct effect but also a spatial spillover effect. Shi et al. (2023) [15] argues that the development of the digital economy not only contributes to the enhancement of urban resilience but also that the impact has a positive spatial spillover effect. Jin Zhaohui et al. (2024) [16] and Zhang Chunmin (2024) [17] find that promoting the development of urban digital economy can significantly improve the economic resilience of cities. Wang et al. (2023) [18] finds that the digital economy improves urban resilience by optimizing the effect of urban layout and promoting the upgrading of urban industrial structure (Chen Shengli et al., 2021) [19]. Wang et al. (2023) [20] also finds that the digital economy improves urban resilience by optimizing the urban layout effect and promoting the upgrading of the urban industrial structure (Chen Shengli et al., 2021) [19], while Wang et al. (2024) [20] finds that the digital economy has a positive impact on regional economic resilience by optimizing the urban layout effect and promoting the upgrading of the urban industrial structure (Chen Shengli et al., 2021) [19]. However, scholars have paid less attention to the impact of green financing on economic resilience. Zhou et al. (2024) [21] avers that green finance could effectively stimulate market vitality and enhance urban economic resilience by playing the functions of capital allocation, market pricing, and risk management. Yao et al. (2023) [22] finds that the impact of green finance on economic resilience comes mainly from industrial upgrading, technological innovation, and scale of development. Cao et al. (2023) [23] analyzes the quantile regression method and finds that green finance plays a key role in enhancing economic resilience regarding scarce or abundant resources.

Based on the analysis presented, the existing literature has explored the topics of the digital economy, green finance, and economic resilience. However, there remains a notable research gap: the interrelationships among these three areas. Consequently, the pathways through which the digital economy influences economic resilience, and the pivotal role of green finance in this process, have not yet been comprehensively examined. Furthermore, current studies predominantly focus on agriculture, export-oriented economies, and regional economic resilience, while largely overlooking the impact of green finance on economic resilience within the context of the digital economy. This study makes several significant contributions. First, it analyzes the relationship among the digital economy, green finance, and economic resilience, specifically from the perspective of green finance, thereby elucidating the influence pathways involved. Second, it empirically assesses the impact of the digital economy on enhancing economic resilience and investigates the mediating role of green finance using a variety of econometric methods. Finally, this study provides a theoretical foundation for future investigations into the interplay among the digital economy, green finance, and economic resilience.

## 2. Theoretical analysis and research hypothesis

### 2.1 The impact of the digital economy on economic resilience

With the development of digital communication technology, the concept of the digital economy was first put forward by Don Tapscott in 1996, who believed that the digital economy is a network system that combines technology, knowledge, and innovation to promote social development. Sun et al. (2004) [24] further re-analyzes the basic features and nature of the digital economy and argues that the digital economy is mainly embodied in information technology. With the development and wide application of digital technologies such as big data and artificial intelligence, the digital economy has continued to expand and deepen globally and has become an important force driving economic growth. The digital economy has not only given rise to new production methods and business models but also has a profound impact on employment structure, consumption patterns, social governance, and other aspects, thereby affecting economic resilience. First, regarding production methods and business models, the digital economy can leverage information technology to improve production methods, foster new industries, and accelerate the innovation of new business models. Yong et al. (2024) [25] avers that under the impetus of the digital economy, the traditional mode of production cannot meet the needs of real economic development, and the innovative, platform-based, green mode of production has begun to accelerate the development of the economic subject not only to meet the needs of the competition to innovate the business model but also to be forced to choose under a competitive environment (Gu Limin et al. 2023) [26], which will lead to the social transformation of the overall business model and innovative mode of production, promoting the economic resilience of the economy. production modes, thereby promoting economic resilience. Through empirical analysis, Qian et al. (2023) [27] finds that production services and business models within the digital economy can enhance regional innovation levels, thereby driving economic growth. Additionally, Xuanyu et al. (2023) [28] notes that the digital economy has transformed production methods through the substitution relationship between data and traditional production factors, promoting the integrated development of the digital and real economies. Second, regarding the employment structure, the development of the digital economy inevitably leads to changes in the skill requirements of workers: low-skilled labor suppliers are gradually eliminated and migrate to the service sector (Acemoglu D et al. 2018) [29]; conversely, high-skilled workers will maintain their advantages and continue to work in high-skilled industries (Acemoglu D et al. 2020) [30] and guide the upgrading of the skills of the new labor force under high income and social demand, but there will not be a drastic change in the total amount of employment in society (Wu Ji-Ying et al. 2024) [31]. This is mainly because digital technology will give rise to new occupations and improve the degree of matching. Third, regarding consumption, the development of the digital economy can overcome the previous limitations of time and space on consumer behavior (Song Baolin, 2024) [32] and give rise to new consumption scenarios (Wang Ye, 2024) [33]. This not only changes consumers’ consumption patterns but also continuously promotes the upgrading of consumer spending and guides production demand driven by changes in consumption. Xiaoxuan et al. (2024) [34] notes that digital industrialization and industrial digitization have not only created new consumer demand but also established new consumption supply models, effectively promoting the expansion and upgrading of consumption, which has positively impacted high-quality economic development. The analysis by Wang Song et al. (2024) [35] shows that while the digital economy promotes consumption upgrading and economies of scale, it also fosters the development of tertiary industries, improves regional industrial structures, and advances the coordinated development of regions, urban and rural areas, and industries. Finally, regarding social governance, Yingying et al. (2024) [36] avers that the digital economy optimizes and coordinates agricultural production layout and urbanization, promoting the intensification and efficiency of production space. This facilitates mutual integration and common development between urban and rural areas regarding planning, layout, and industrial development, thereby reshaping the social governance structure and regional spatial patterns. Hui Ning et al. (2023) [37] argues that the digital economy improves social governance methods, enhances fairness and efficiency in governance, increases the quality of public services, and facilitates the precise connection and inclusiveness of regional social services, thus perfecting the balanced allocation of basic public service resources (Li Chenglong, 2024) [38]. According to the analysis by Ai Lin et al. (2023) [39], the participation of the digital economy in social governance can effectively strengthen the free flow of production factors within society. This is primarily due to technological advancements that reduce working hours and blur the boundaries of work locations through improved production methods, thereby allocating social resources efficiently. Therefore, the development of the digital economy has given rise to new economic forms, proposed new requirements for workers’ occupational skills, improved the allocation efficiency of social resources, and contributed to the maintenance of economic vitality and resilience. Based on the above analysis, this study proposes the following hypothesis:

Hypothesis 1: The digital economy helps to enhance economic resilience.

### 2.2 The impact of green finance on economic resilience

Green finance focuses on environmental protection and sustainable development, and has become an important force supporting the development of green, low-carbon, and circular economies. Green financing affects economic resilience through resource allocation, technological innovation, and risk management. First, by optimizing resource allocation, green finance guides the flow of capital into the production and living activities of environmental protection and low-carbon projects (Peng Diyun et al., 2024) [40], advancing the industrial structure in the direction of decarbonization, which accords with the future direction of the economy and accelerates the structural adjustment of the economy to enhance its future resilience. Through an empirical analysis, Yingtao et al. (2024) [41] finds that green finance positively impacts urban economic efficiency and ecological benefits by optimizing resource allocation, promoting technological innovation, aggregating productive service industries, and attracting new enterprises into the market. This, thus, facilitates high-quality development of urban economies. Wentao et al. (2023) [42] notes that the resource allocation function of green finance should be fully utilized to support green development, thereby promoting the transformation of regional industrial structures towards ecological sustainability and achieving the sustainable development of regional green economies. Second, green finance promotes the innovation of green technology by supporting the development of low-carbon technology and clean energy (Yu Shaolong et al., 2024) [43], enhancing the adaptive and transformational capacity of the economy, promoting an advanced and rationalized industrial structure, and improving the ability of the economic system despite uncertain risk shocks to enhance the resilience of the economy. Chunxi et al. (2024) [44] argues that green finance enhances the resilience of urban economies by alleviating financing constraints, reducing financing costs, and serving as a signaling mechanism that promotes technological innovation. This strengthens the recovery, adaptability, and transformation capabilities of urban economies. Through an empirical analysis, Bo et al. (2022) [45] finds that green finance can promote technological innovation in enterprises by broadening financing channels, alleviating information constraints, and improving risk management, thereby positively impacting the high-quality development of the economy. Finally, green finance helps identify and manage environmental risks, effectively disperses risks (Zhou Chunxi et al., 2024) [44], reduces the potential losses of financial institutions and enterprises during complex environmental changes, improves the operational efficiency of enterprises, improves quality and efficiency, and enhances the overall economic resilience of society. Ying et al. (2024) [46] notes that a green finance market can provide investors with financial products that cater to different risk preferences, thereby offering stable long-term funding support for technological innovation. This promotes technological upgrades and accelerates the adjustment and optimization of economic structures. Wang et al. (2021) [47] argues that green finance can internally support enterprise financing through resource allocation and risk management, while enhancing companies’ ability to identify innovation risks, reducing the difficulties enterprises face in securing financing during the innovation process, and strengthening their capacity to diversify risks, ultimately promoting regional economic competitiveness. Therefore, the development of green finance enhances the vitality of the economy, innovation ability, and management ability to cope with risk, which improves the resilience of economic development. Based on the above analysis, this study proposes the following hypothesis:

Hypothesis 2: Green finance helps to enhance economic resilience.

### 2.3 The impact of the digital economy on economic resilience through green finance

The development of the digital economy has significantly transformed production and consumption methods, enhancing the universality of financial services. This has alleviated the financing constraints faced by many small and medium-sized enterprises (SMEs) and green technology innovations. Furthermore, by leveraging its digital technology advantages, it has improved resource allocation efficiency, innovative green financial products, and enhanced risk management capabilities, thereby meeting the needs of numerous potential fund seekers in the long-tail effect and driving the growth of green finance. Tan Xiaofen et al. (2024) [48] argues that digital finance is a manifestation of the digital economy in the financial sector and that the level of digital economy development influences the growth of digital finance. Shefang et al. (2024) [49] finds that the digital economy not only expands the scale of financial development but also reduces costs in the financial industry, improving the efficiency of financial resource allocation, which promotes the overall development of finance. Additionally, the development of green finance boosts green total factor productivity, with a spatial spillover effect (Yu Shaolong et al., 2024) [43], thereby driving overall economic growth. Tao et al. (2024) [50] suggests that the development of digital finance can alleviate financing difficulties and improve credit accessibility, thereby fostering innovation and entrepreneurship and promoting inclusive regional economic growth. Simultaneously, the clear green industry-oriented objectives of green finance have indirectly reshaped green consumption patterns and incentivized enterprises to adopt green production methods, leading to holistic improvement and economic and societal development. Wang et al. (2024) [51] argues that green finance, which relies on national policies and future development needs, can promote industrial structure upgrading and stimulate economic growth. Based on an empirical analysis, Xiaoke et al. (2024) [52] emphasizes the need to continuously improve the mechanisms through which green finance supports industrial structure optimization, strengthening its incentive effects on enterprise innovation to foster better economic development. Therefore, the synergistic role of digital economy and green finance can effectively promote economic development and enhance economic growth and resilience. Based on the above analysis, this study proposes the following hypotheses:

Hypothesis 3a: Green finance has a mediating effect on the digital economy and economic resilience.
Hypothesis 3b: Green finance initiatives enhance economic resilience by stimulating corporate innovation, and research and development (R&D), fostering sustainable growth and mitigating the risks associated with economic shocks.
Hypothesis 3c: Green finance strengthens economic resilience by reducing environmental pollution, thereby mitigating ecological risk and fostering sustainable economic stability.

## 3. Model setup and data description

### 3.1 Benchmark model construction

This study conducts empirical analyses based on panel data of 30 provinces and cities in China from 2011 to 2023 and constructs the model shown in Eq (1):

$$Resi_{it}=\alpha_{0}+\alpha_{1}D_{it}+\alpha_{2}X_{it}+\mu_{i}+\delta_{t}+\varepsilon_{1it},$$ (1)

where subscript *i* denotes province, *t* denotes year, the explanatory variable *Resi*_*it*_ is the provincial level of economic resilience in province *i* in year *t*, the core explanatory variable *D*_*it*_ is the level of digitization in province *i* in year *t*, *X*_*it*_ is a control variable, *μ*_*i*_ is an individual fixed effect, *δ*_*t*_ is a time fixed effect, and *ε*_1*it*_ is a random disturbance term.

### 3.2 Intermediary effect modelling

Combined with the above analysis, this study argues that the digital economy affects economic resilience and green finance plays a mediating role in the impact of the digital economy on economic resilience. Therefore, this paper selects green finance as the intermediary variable to empirically analyze the relationship between the digital economy, green finance and economic resilience, and the specific intermediary model is set as shown in Eqs (2) and (3):

$$M_{it}=\beta_{0}+\beta_{1}D_{it}+\beta_{2}X_{it}+\mu_{i}+\delta_{t}+\varepsilon_{2it}$$ (2)

$$Resi_{it}=\gamma_{0}+\gamma_{1}M_{it}+\gamma_{2}D_{it}+\mu_{i}+\delta_{t}+\varepsilon_{3it},$$ (3)

where *M*_*it*_ is the mediator variable, denoting the green financial development index of province *i* in year *t*, and *ε*_2*it*_、*ε*_3*it*_ is the random perturbation term is the random perturbation term.

### 3.3 Spatial spillover effect model construction

Considering the differences in the economic development of China’s regions, this paper adopts a spatial econometric model to study the relationship between the digital economy, green finance, and economic resilience, and the specific model settings are shown in Eqs (4)–(6):

$$Resi_{it}=\alpha_{0}+\rho_{1}W\times Resi_{it}+\delta_{1}W\times D_{it}+\alpha_{1}D_{it}+\delta_{c}W\times X_{it}+a_{c}X_{it}+\mu_{i}+\delta_{t}+\varepsilon_{1it}$$ (4)

$$Resi_{it}=\alpha_{0}+\rho_{1}W\times Resi_{it}+\alpha_{1}D_{it}+a_{c}X_{it}+\mu_{i}+\delta_{t}+\varepsilon_{1it}$$ (5)

$$\{\begin{array}{c} Resi_{it}=\alpha_{0}+\alpha_{1}D_{it}+\alpha_{c}X_{it}+\mu_{i}+\delta_{t}+\varepsilon_{1it}\\ \varepsilon_{1it}=\lambda W_{\varepsilon_{1it}}+\upsilon_{1it} \end{array},$$ (6)

where (4) ~ (6) represent the spatial Durbin model (SDM), spatial autocorrelation model (SAR), and spatial error model (SEM), respectively. Where *ρ*_1_ represents the spatial autoregressive coefficient, W represents the spatial weight matrix,*δ*_1_ represents the spatial interaction term coefficient of the digital economy, *δ*_*c*_ represents the spatial interaction term coefficient of the control variables, and λ represents the spatial interaction term coefficient of the random perturbation term. The remaining variables are consistent with Eq (1). When λ = 0, it is the SDM model shown in Eq (4); when λ = 0, *δ*_1_ = 0 and *δ*_*c*_ = 0, it is the SAR model shown in Eq (5), and when *ρ*_1_ = 0, *δ*_1_ = 0 and *δ*_*c*_ = 0, λ≠0, it is the SEM model shown in Eq (6). In this study, an applicable model was selected for spatial spillover effect analysis based on subsequent test results.

### 3.4 Variable selection and data sources

#### 3.4.1 Description of variables

*(1) Explained variables*. Economic resilience (Resi) was selected as an explanatory variable. Since economic resilience cannot be reflected by ready-made indices, this study draws on the index construction method of previous scholars (Cai Yongmei et al., 2024) [53], and constructs the economic resilience index through the entropy weighting method from the four dimensions of the risk, stability, circulation, and innovation indices. The construction indices are listed in Table 1.

**Table 1:** System of economic resilience indicators.

Dimension	Indicators	Indicator metrics	Symbolic
Risk index	Urban registered unemployment rate (%)	(Number of urban registered unemployed/(Total number of employed persons + number of urban registered unemployed))*100%, reflecting regional employment status	Negative
	Direct economic losses from natural disasters ($ million)	Reflecting regional damage due to natural disasters	Negative
	Industrial wastewater discharge per unit area (tonnes/km^2^)	Industrial wastewater discharges/area of land, reflecting the ecological impacts of the area	Negative
	Foreign trade dependence (%)	(Total imports/GDP)*100%, reflecting the degree of regional dependence on imports.	Negative
Stability index	GDP per capita (10,000 yuan/person)	GDP/total population, reflecting the overall level of economic development of the region.	Positive
	Share of social security expenditure (%)	Social security expenditure/fiscal expenditure*100%, reflecting regional social security inputs	Positive
	Percentage of people insured against unemployment (%)	Number of persons insured against unemployment/number of persons employed at the end of the year * 100%, reflecting the social security status of unemployed persons in the region	Positive
	Per capita investment in fixed assets (yuan per person)	Total investment in fixed assets/total population, reflecting the situation of investment in fixed assets in the region.	Positive
	Retail sales of consumer goods per capita (10,000 yuan/person)	Total retail sales of consumer goods/total population, reflecting the level of regional consumer demand and economic prosperity	Positive
Liquidity index	Railway density (km/km^2^)	Railway mileage/land area of the region, reflecting the construction of the regional railway network	Positive
	Road density (km/km^2^)	Road mileage/district land area, reflecting district road network construction	Positive
	Balance of loans to financial institutions (billions)	Reflecting the borrowing or financing of enterprises from banks and the liquidity of regional currencies	Positive
	Internet ports per 10,000 people (ports per 10,000 people)	Reflecting the level of information and communication, as well as the ability to circulate information in regional cyberspace	Positive
	Employed population in railway transport (persons)	Reflecting the service guarantee capacity of railway logistics	Positive
Innovation index	Expenditure on education (%)	Education expenditure/fiscal expenditure*100%, reflecting the level of regional investment in education	Positive
	Share of science and technology expenditures (%)	S&T expenditure/fiscal expenditure*100%, reflecting the level of regional investment in S&T	Positive
	Number of R&D personnel (persons)	Reflecting regional innovation capacity	Positive
	Share of private enterprise and self-employment (%)	Private sector employment + self-employment)/total employment*100%, reflecting the dynamism of regional economic development and entrepreneurship	Positive

*(2) Explanatory variables*. Since the digital economy (Digf) involves multiple aspects and there is currently no single indicator that can be effectively measured for the time being, this study draws on the methodology of previous scholars (Jingshan Gu et al., 2024) [54], and looks at the three dimensions of Innovation Drive, Level of Development and Industrial Enhancement (see Table 2).

**Table 2:** Digital economy indicator system.

Dimension	Indicators	Indicator metrics	Symbolic
Innovation drive	Digital infrastructure	Number of Internet broadband access subscribers	Positive
	Digital environment	All-society fixed-asset investment in the information transmission, computer services and software industry	Positive
	Elements of digital innovation	Expenditures on science and technology	Positive
Level of development	Level of digital outputs	Number of digital economy-related patents per 10,000 people	Positive
	Digital technology penetration	Websites per 100 businesses	Positive
	Number of relevant employees	Employed persons in information transmission, software and information technology services/employed persons in urban units	Positive
Industrial enhancement	Digital Inclusive finance index	Peking University Digital Inclusive Finance Index	Positive
	Industrial digitization	Number of computers per 100 persons in industrial enterprises	Positive
	Industry digital outputs	E-commerce turnover of industrial enterprises and its share in the main business income of industrial enterprises	Positive

Green finance (greenf) also involves many aspects, and there is no single indicator that can effectively measure its development; therefore, this study draws on the methodology of previous scholars (Xiao Gu et al.,2023) [55], and constructs four dimensions: green credit, green investment, green insurance, and government support, as shown in Table 3. Supports were constructed and the specific constructed indicators are listed in Table 3.

**Table 3:** Green finance indicator system.

Dimension	Indicators	Indicator metrics	Symbolic
Green credit	Proportion of high energy consumption industry interest expenditure	Interest expenditure of six high energy consuming industrial industries/Total industrial interest expenditure	Negative
Green investment	Proportion of environmental pollution control investment in GDP	Environmental pollution control investment/GDP	Positive
Green insurance	Depth of agricultural insurance	Agricultural insurance income/Gross agricultural output value	Positive
Government support	Proportion of environmental protection expenditure	Environmental protection expenditure/General budget expenditure	Negative

*(3) Control variables*. Based on existing literature, the control variables selected in this study are as follows: Human capital level (std): expressed as the logarithm of the number of undergraduates enrolled in regional colleges and universities (Lu Xianxiang et al., 2024) [56]; industrial structure (ind): measured by the ratio of the value added of the tertiary industry to that of the secondary industry (Lu Xianxiang et al., 2024) [56]; economic level (lngdp): expressed as the natural logarithm of GDP (Liu Li et al., 2023) [57]; openness to the outside world (open): expressed as the logarithm of the amount of foreign investment actually used in the current year (Liu Li et al., 2023) [57]; and population density (popdensity): measured using the number of resident population per square kilometer (Wei Feng et al., 2023) [58]. The degree of economic activity dispersion (disper) was measured by the actual urban road area per capita (Mao Fengfu et al., 2022) [59].

#### 3.4.2 Data sources

Based on the principles of data availability and science, this paper selects data from 30 Chinese provinces (excluding Hong Kong, Macao, Taiwan, and Tibet) from 2011–2023 as the research sample. For any missing data, this study adopted interpolation using the average growth rate. The descriptive statistics of the main variables are presented in Table 4.

**Table 4:** Descriptive statistics.

Variable	Obs	Mean	Std. Dev.	Min	Max
Resi	390	2.29	1.538	0.269	7.926
digf	390	0.14	0.109	0.002	0.736
greenf	390	0.196	0.121	0.06	0.944
ind	390	1.331	0.737	0.527	5.244
lngdp	390	9.813	0.91	7.042	11.772
popdensity	390	340.358	315.413	3.632	1334
open	390	83.626	93.301	0.032	1016.471
std	390	93.024	56.571	4.5	282.33
disper	390	16.270	5.070	4.04	28

## 4. Empirical analysis

### 4.1 Benchmark regression

Before empirically testing the relationship between digital technological innovation and urban economic resilience, it is necessary to determine whether the econometric model established in this study has spurious regression or pseudo-regression problems that require a test of the smoothness of the panel data. From the Table 5, we can see the results of the Hausman test show that the original hypothesis is rejected at the 1% significance level, and then this study uses a fixed-effects model to conduct a benchmark regression.

**Table 5:** Hasuman test and Ramsey RESET.

	Hausman Test	Ramsey RESET test
Test result	chi2(7) = 138.09	F(1, 340) = 2.21
P-value	0.000	0.138

We also performed the Ramsey Regression Equation Specification Error Test (RESET) to model misspecification. The Ramsey RESET is a diagnostic tool used in regression analysis to detect specification errors in a linear regression model. Specifically, it assesses whether important explanatory variables have been omitted, whether the functional form of the model is incorrect, or whether there is an overall model misspecification. The test involves adding polynomial terms or other transformations of the predicted values to the original regression equation and then testing the joint significance of these additional terms. If these terms are statistically significant, this suggests that the original model fails to capture certain aspects of the data, indicating misspecification. Essentially, the Ramsey RESET evaluates whether nonlinear combinations of independent variables have explanatory power not accounted for in the model, signaling potential issues such as omitted variables or incorrect functional forms. From the Table 5, we can see the results of the Ramsey RESET show that the original hypothesis cannot be rejected at the 10% significance level, it suggests that there are no important explanatory variables have been omitted.

The regression results are shown in Table 6, where column (1) presents the regression results with only the core explanatory variable D and column (2) presents the fixed effects estimation results controlling for city and year. The regression coefficients for the digital economy development index (D) are 9.097 and 5.282, respectively, and both pass the 1% significance test. Column (3), conversely, controls for year fixed effects and includes control variables to consider the impact of the level of digital technological innovation on the economic resilience of cities across provinces in the same year. Column (4) controls for city and year double fixed effects while adding the control variables. From the results, it can be seen that there was always a positive correlation between the two at the 1% confidence level, which means that the results of the regression coefficients were reliable. The results are fully consistent with the theoretical expectations: an increase in the level of digital technological innovation significantly improves the economic resilience of the city. Digital technological innovation is conducive to the "elasticity" of urban subjects to adapt to changes in the external environment, and to perceive and adapt to environmental changes by continuously adjusting the way of organizational operation after the impact. Simultaneously, it promotes the integration of digital technology and the real economy, gives the economy a new advantage in development, and mitigates the impact of external shocks on the city’s economy. Additionally, it helps improve the economic resilience of cities and helps enhance the resilience of a city’s economy. Further, from the estimation results of the control variables under the double fixed-effects model, it can be seen that the regression coefficients of industrial structure (ind), economic scale (lngdp), and population density are significantly positive. Hence, indicating that the more developed the tertiary industry, the larger the economic scale, and the more densely populated the province, and the market scale can ebe utilized fully. Further, it accelerates the dissemination of information through the spillover of technological knowledge and the reorganization of factors to optimize the resource allocation efficiency and improve the urban economic resilience, efficiency, and improve urban economic resilience. The degree of dispersion of economic activities (disper) is significantly negatively correlated with economic resilience because an overly extensive road network may lead to excessive dispersal of economic activities, weakening the agglomeration effects between businesses and industries. This limits the exchange of knowledge and technology, reduces innovation efficiency, and improves economic competitiveness and resilience.

**Table 6:** Baseline regression result.

	(1)	(2)	(3)	(4)
	Resi	Resi	Resi	Resi
Digf	9.097***	5.282***	5.344***	4.802***
	(0.236)	(0.329)	(0.482)	(0.431)
ind			-0.0735	0.019
			(0.084)	(0.089)
lngdp			0.959***	0.992***
			(0.126)	(0.204)
popdensity			0.002*	0.004***
			(0.001)	(0.001)
open			-0.000	-0.000
			(0.000)	(0.000)
std			0.001	-0.004***
			(0.002)	(0.001)
disper			-0.003	-0.026***
			(0.010)	(0.009)
_cons	1.020***	4.156***	-7.662***	-10.581***
	(0.135)	(0.097)	(1.816)	(2.103)
city_id	NO	YES	YES	YES
year	NO	YES	NO	YES
N	390	390	390	390
R^2^	0.7174	0.9742	0.9656	0.9786

Standard errors in parentheses, * *p* < 0.1

** *p* < 0.05

*** *p* < 0.01

### 4.2 Endogenous problem treatment

There may be a mutual causal relationship between digital economy development and provincial economic resilience: cities with higher provincial economic resilience tend to be more inclined to engage in digital technology innovation, and the higher city economic resilience may be the cause rather than the effect of digital technology innovation. Therefore, this study adopted the following method to rule out the endogeneity problem.

The historical evolution of Internet access technology in China began with dial-up (PSTN), followed by ISDN, ADSL, and, ultimately, the widespread adoption of fiber broadband. Consequently, the development of internet technology is inherently linked to the proliferation of fixed-line telephony. Regions with historically high penetration rates of fixed-line telephones are likely to exhibit higher Internet penetration rates. Before fixed-line telephones became common, the primary means of communication was through postal systems. Post offices played an instrumental role in the installation of fixed-line telephony, and their distribution influenced the allocation of telephone lines, consequently affecting the prioritization of Internet access. Thus, the postal system’s influence on communication practices has affected the broader adoption and development of the Internet. Hence, the number of fixed-line telephones and post offices is suitable as an instrumental variable for digital economy development because they satisfy the relevance criterion.

Furthermore, the historical numbers of fixed-line telephones and post offices have largely lost their influence on provincial economic resilience, given the weakened association between them. Thus, after accounting for other control variables, using historical numbers of fixed-line telephones and post offices as instrumental variables fulfilled the exogeneity requirement to a considerable extent. Based on this reasoning, this study employs the historical number of fixed-line telephones per hundred people and the number of post offices per million people in 1998 as instrumental variables for the development of the digital economy.

Given that the dataset in this study is a balanced panel, directly using the historical number of fixed-line telephones and post offices as instrumental variables poses challenges in implementing a fixed-effects model. To address this issue, following the methodology of Nunn and Qian (2014) [60], this study constructs interaction terms between the historical number of fixed-line telephones per hundred people and the number of post offices per million people in 1998 (both time-invariant), and the number of Internet broadband access ports (which vary over time) to serve as instrumental variables for digital economy development.

Based on the statistical output of the instrumental variable (IV) regression diagnostics (Table 7), we can interpret the results collectively as follows: The under-identification test, with an Anderson canonical correlation LM statistic of 226.624 and a p-value of 0.0000, rejected the null hypothesis of under-identification. This means that the instruments were significantly correlated with the endogenous variables, satisfying the relevance condition necessary for valid instruments. The weak identification test further supports this, as the Cragg-Donald Wald F statistic is 235.811, which is substantially higher than all Stock–Yogo critical values (the highest being 19.93 for a 10% maximal IV size). This confirms that the instruments are strong and that weak identification is not a concern. Finally, the over-identification test yielded a Sargan statistic of 0.598 with a p-value of 0.4394, leading us to fail to reject the null hypothesis, that the instruments are valid and uncorrelated with the error term. This finding suggests that the instruments satisfy the exogeneity condition. Overall, these diagnostic tests collectively confirm that the instruments used in the IV regression are both relevant and valid, thus providing confidence in the consistency and reliability of the estimated coefficients.

**Table 7:** Endogeneity problem processing results.

Variable	IV
Digf	6.248***	
Control	YES	
Id fixed	YES	
Year fixed	YES	
Underidentification test (Anderson canon. corr. LM statistic):	226.624 (p = 0.000)	
Weak identification test (Cragg-Donald Wald F statistic):	235.811 (critical = 19.93)	
Overidentification test (Sargan statistic):	0.598 (p = 0.4394)	
N	390	
R2	0.9932	

* *p* < 0.1

** *p* < 0.05

*** *p* < 0.01(same below)

### 4.3 Mediating effect

This study uses the mediating effect of green finance on the relationship between the level of economic resilience and digital economy development. Sobel–Goodman mediation tests were used to detect the mediating mechanism effect. As shown in Table 8, the p-values of the Sobel, Aroian, and Goodman tests are less than 0.01, hence the mediating effect of the mediating variable between the independent and dependent variables is statistically significant. These tests are all used to assess the strength of the mediating effect and its statistical significance; specifically, the Sobel test is a common method of testing the statistical significance of the mediating effect by assessing whether the mediating variable carries information regarding the effect of the independent variable on the dependent variable. A significant p-value of the Sobel test implies that the mediating effect is significant, thus supporting the idea that the mediating variable plays a key role in the relationship between the independent and dependent variables. The Aroian test is a variant of the Sobel test that provides a more conservative p-value considering greater uncertainty and bias adjustments. The significant results from the Aroian test further strengthen the significance of the mediating effect. The Goodman test is another variant of the Sobel test, and typically produces a more generous confidence interval. The Goodman test also showed significance, providing additional confirmation of the significance of the mediation effect. Hence, we conclude with a high degree of confidence that the mediating variable plays a key role in mediating between the independent and dependent variables. These results support the mediation model hypothesis that the independent variable influences the dependent variable through a mediating variable. This strong statistical evidence suggests that the mediating variable is not only present, but also important in the relationship between the variables. Therefore, our findings support H3, that green finance has a mediating effect between the digital economy and economic resilience.

**Table 8:** Mediating effect test results.

Sobel-Goodman Mediation Tests				
	Est	Std_err	z	P>z
Sobel	4.628	0.355	13.020	0.000
Aroian	4.628	0.356	13.010	0.000
Goodman	4.628	0.355	13.029	0.000
Indirect, Direct, and Total Effects				
	Est	Std_err	z	P>z
a_coefficient	0.723	0.042	17.045	0.000
b_coefficient	6.401	0.317	20.173	0.000
Indirect_effect_aXb	4.628	0.355	13.020	0.000
Direct_effect_c’	7.274	0.351	20.746	0.000
Total_effect_c	11.902	0.379	31.382	0.000
Proportion of total effect that is mediated:			0.389	
Ratio of indirect to direct effect:			0.636	
Ratio of total to direct effect:			1.636	

### 4.4 Spatial measurement regression results

#### 4.4.1 Spatial autocorrelation test

Theory and practice show a spatial clustering phenomenon in provinces with similar levels of economic resilience in China. The reason for this is the obvious spatial spillover effect of industrial diversification, industrial agglomeration, the digital economy, and other factors on economic toughness. Therefore, whether there is a spatial spillover effect on provincial economic resilience in promoting the digital economy development deserves further investigation.

To verify whether there is spatial autocorrelation between digital economy development and economic resilience in different provinces, firstly, the spatial autocorrelation of economic resilience of the explanatory variables of each statistical province in 2011–2023 is tested by the global Moran’s test, and our results are shown in Table 9. The spatial weight matrix constructed in this study, is a spatial economic geographic weight matrix, made up of latitude, longitude, and GDP per capita: $W-W_{d}diag\left(\frac{X_{1}}{X},\frac{X_{2}}{X},\ldots ,\frac{X_{i}}{X}\right)$, where *W*_*d*_ is the geographic inverse distance matrix, *X*_*i*_ is the GDP per capita of the region of province *i* from 2011–2023, and X is the sum of the mean value of the gross regional product of province *i* from 2011–2023. From Table 9, we can see that the indices under the spatial weight matrix all reach a significance level of 1%, indicating that the development of the digital economy and the economic resilience of each province each have a spatial dependence, while the indices are all greater than 0, indicating that the development of the digital economy and the provincial economic resilience have a spatial positive correlation.

**Table 9:** Global Moran index.

year	Resi	digf	greenf
2011	0.455***	0.249***	0.362***
2012	0.445***	0.230***	0.365***
2013	0.428***	0.193***	0.359***
2014	0.410***	0.244***	0.355***
2015	0.407***	0.170***	0.355***
2016	0.419***	0.225***	0.339***
2017	0.436***	0.247***	0.323***
2018	0.436***	0.203***	0.293***
2019	0.443***	0.184***	0.340***
2020	0.421***	0.157***	0.342***
2021	0.430***	0.134**	0.345***
2022	0.408***	0.157***	0.328***
2023	0.363***	0.148***	0.326***

**Fig 1:**
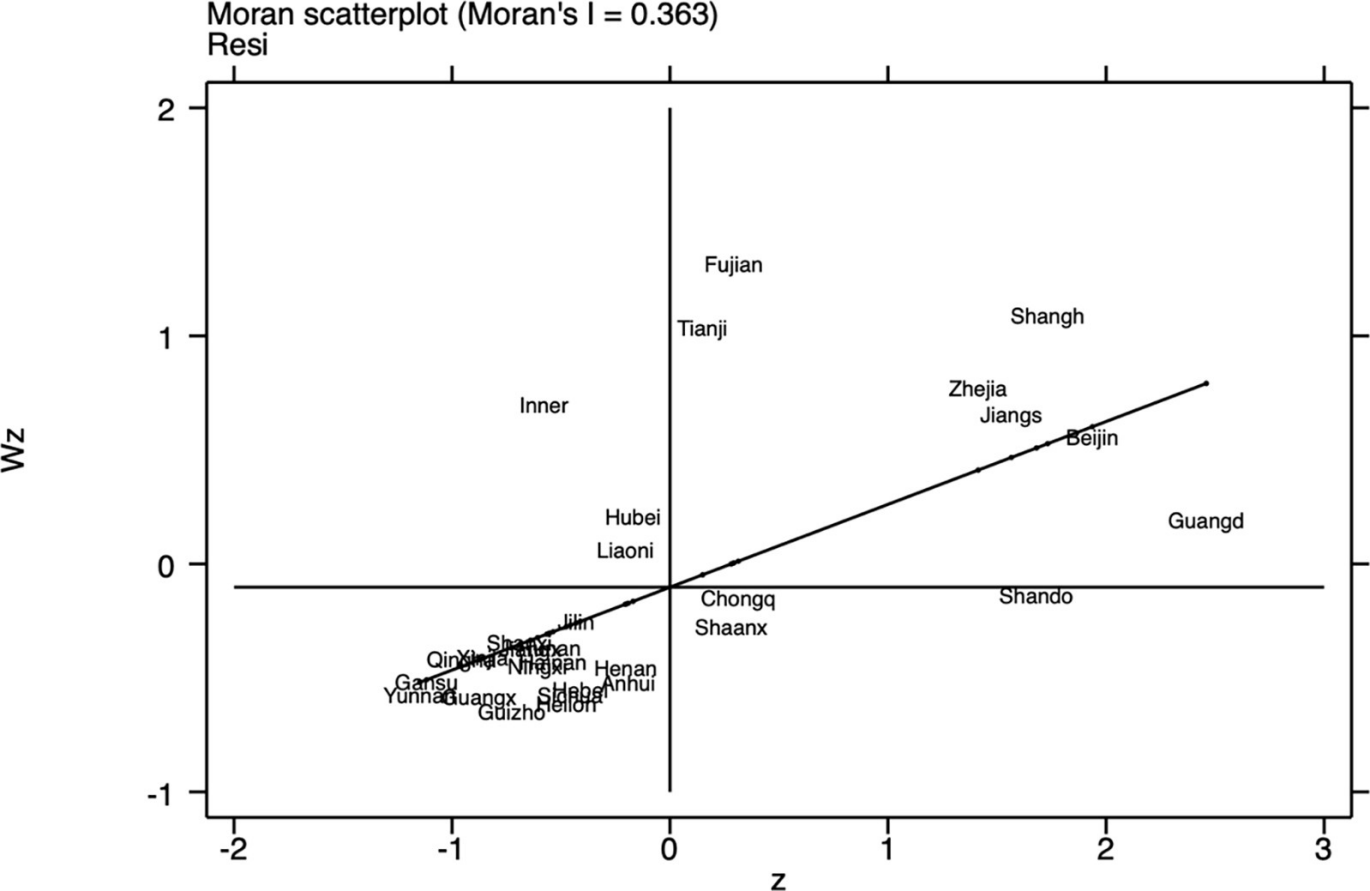
Scatter plot of Moran index of Provincial resilience in 2023.

**Fig 2:**
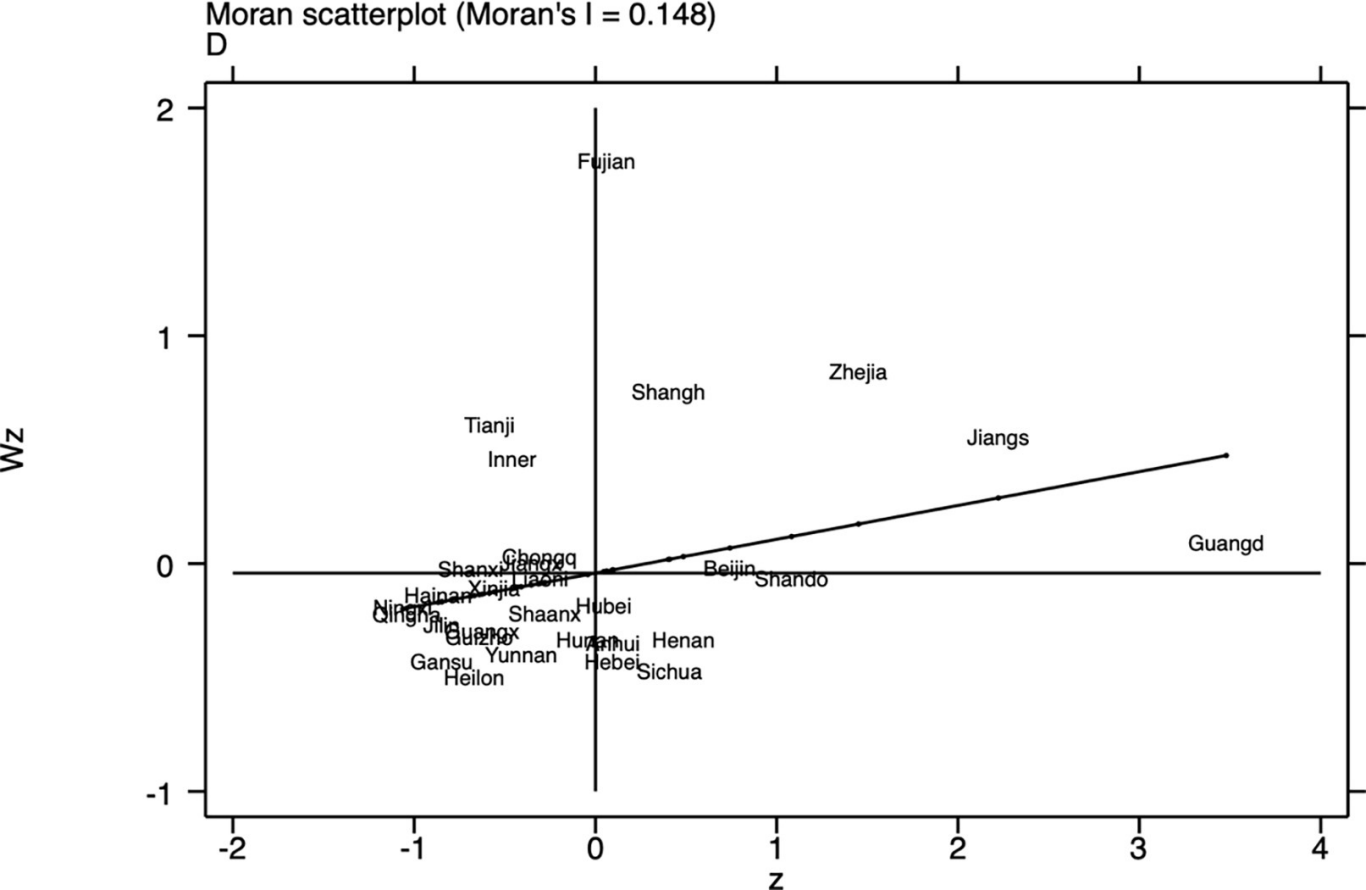
Scatter plot of Moran index of Provincial digital economy development index in 2023.

**Fig 3:**
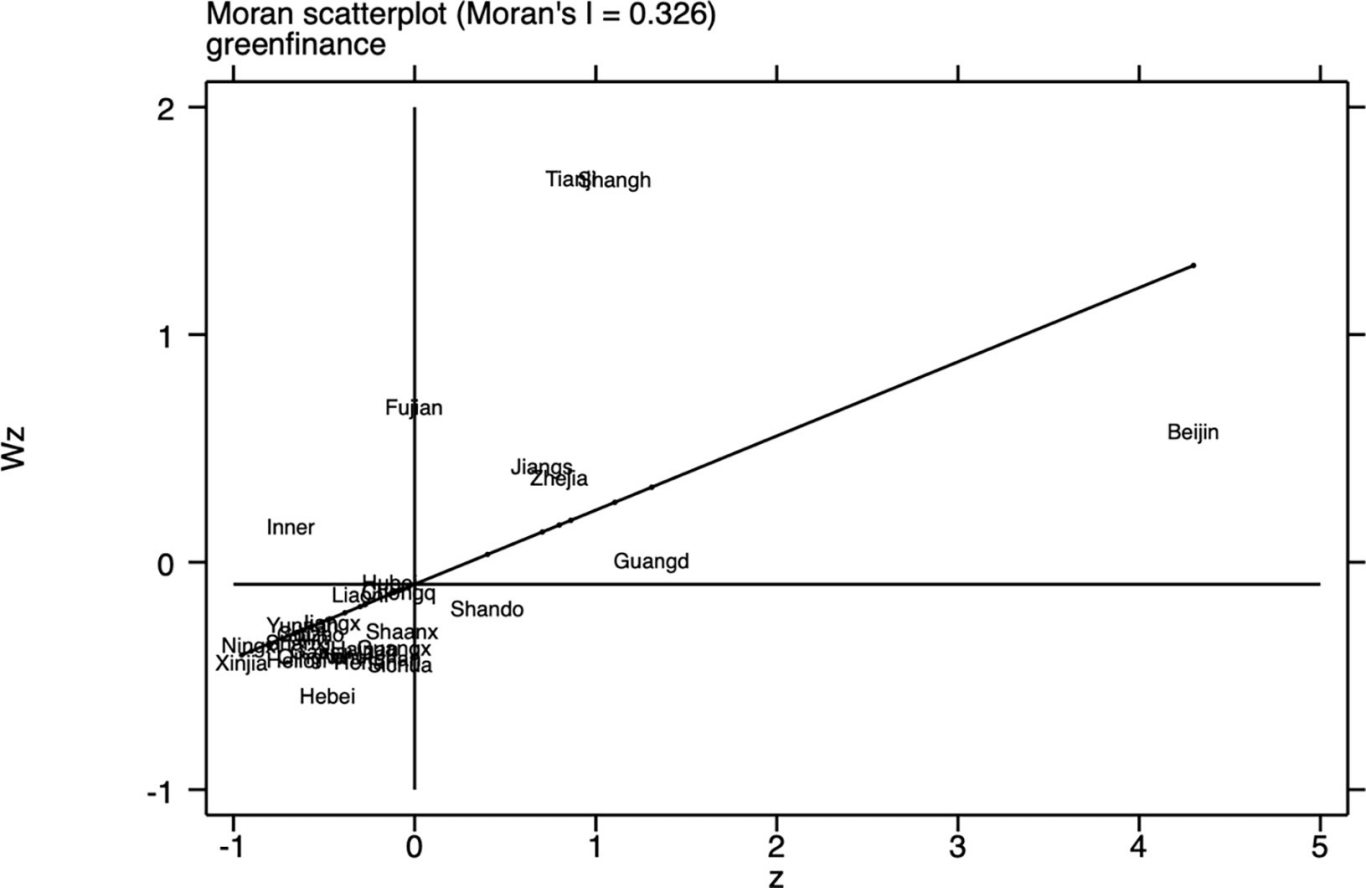
Scatter plot of Moran index of Provincial green finance index in 2023.

The Moran index for 2023 (Figs 1–3) shows that there is a clear spatial correlation between economic resilience, digital economy development, and green finance across China’s provinces, implying that the performance of certain provinces in these three areas may be influenced by the performance of their geographic neighbors and that a stronger digital economy development in one province is likely to be demonstrated by its neighbors in a similarly strong development trend.

#### 4.4.2 Results of empirical spatial panel regression

To select an appropriate spatial econometric model, we conducted LR, LM, Wald, and Hausman tests (see Table 10). In examining the impact of digital economy development on economic resilience, we find that in Model 1, the p-values of R-LM(lag), Wald_lag, Wald_error are 0.949, 0.683, and 0.148, respectively; in Model 2, the p-values of LM(lag) and Wald_error are 0.260 and 0.429, respectively; and in Model 3, the p-values of R-LM(lag) and Wald_lag are 0.490 and 0.396, respectively. None of these results are significant at the 90% significance level, while all other variables are significant. Hence, we tentatively infer that the Spatial Durbin Model (SDM) is superior to both the Spatial Error Model (SEM) and the Spatial Lag Model (SLM). Therefore, after performing the Hausman test, we chose the SDM for our analysis, which contained both time and spatial fixed effects.

**Table 10:** Test results of spatial econometric model.

	Model 1		Model 2		Model 3	
	Statistic	p-value	Statistic	p-value	Statistic	p-value
Moran’s I	9.595***	0.000	7.678***	0.000	10.975***	0.000
LM(error)	80.590***	0.000	50.659***	0.000	105.743***	0.000
R-LM(error)	64.676***	0.000	52.241***	0.000	83.063***	0.000
LM(lag)	15.918***	0.000	1.271	0.260	23.156***	0.000
R-LM(lag)	0.004	0.949	2.854*	0.091	0.476	0.490
Wald_lag	3.95	0.6832	21.68***	0.001	6.25	0.3958
Wald_error	10.80	0.1475	6.99	0.4295	19.71***	0.0062
LR_lag	87.90***	0.000	54.46***	0.0000	142.56***	0.000
LR_error	109.16***	0.000	60.37***	0.0000	168.17***	0.000
Hausman	71.40***	0.000	27.12**	0.012	32.81***	0.005

When testing the impact of the digital economy on economic resilience (Table 11), we found that digital economy development was positively correlated with economic resilience in the provinces. Specifically, a 1% increase in digital economy development is associated with an 8.193% increase in economic resilience, verifying hypothesis H1. In addition, the coefficient of the control variable GDP is positive, indicating a positive correlation between economic development and economic resilience and that the higher the budget, the better the economic development. This may be due to the shift in Chinese government spending from the general public budget to public investment, scientific and technological innovation, and environmental protection, which contribute to improved infrastructure, increased income, and social welfare. GDP growth usually reflects the expansion of the economy’s size and increased output, allowing the government and enterprises to have more resources to invest in critical infrastructure and services, which enhances society’s overall resilience to risk and improves economic resilience. In addition, for every 1% increase in population density, economic resilience increases by 0.0002%, which is attributed to the flexibility of the labor market, which is typically more flexible and diverse in areas with higher population densities. Such a market structure makes it easier for individuals to find new job opportunities after losing their jobs, thereby reducing the impact of economic downturns. Increased population density enhances the economic resilience of a region in several ways by increasing the efficiency of resources and services, promoting the clustering of economic activities, strengthening social capital and networks, improving the targeting of policy inputs, and increasing the flexibility of the labor market.

**Table 11:** Results of spatial Durbin regression.

	Model (1)		Model (2)		Model (3)	
	coefficient	p-value	coefficient	p-value	coefficient	p-value
greenf					4.523***	0.000
Digf	8.193***	0.000	0.336***	0.000	6.684***	0.000
ind	0.234***	0.000	0.064***	0.000	-0.061	0.430
lngdp	0.363***	0.000	0.034***	0.000	0.213***	0.003
popdensity	0.0002**	0.031	0.0001***	0.000	-0.0002*	0.079
open	0.002***	0.000	0.00005**	0.041	0.0018***	0.000
std	-0.007***	0.000	-0.0005***	0.000	-0.005***	0.000
disper	-0.018***	0.008	-0.0024***	0.000	-0.008	0.221
R^2^	0.780		0.8242		0.8532	

In examining the impact of digital economic development on green finance, it was found that a 1% increase in digital economic development was associated with a 0.336% increase in green finance, indicating a positive correlation between digital economic development and green finance. The higher the level of digital economic development, the higher the green finance, and vice versa, thus verifying H2. This is due to the creation of new financing channels, the development of the digital economy provides new financing channels and tools for green finance, such as green bonds, green funds, etc., which make it easier for funds to flow to the enterprises that support environmental protection and green projects, and further strengthen the ecosystem of green finance. Simultaneously, the development of the digital economy has helped increase the transparency of financial markets, making it easier for investors to access information on corporate environmental behavior and sustainable development practices. This increased transparency increases the attractiveness of green financial products and drives capital flows to environmentally friendly projects. Among the control variables, industrial structure and green finance are positively correlated. Specifically, for every 1% increase in the ratio of the tertiary to the secondary industries, green finance increases by 0.064%, owing to the environmentally friendly characteristics of the tertiary industry. Tertiary industries usually have lower levels of energy consumption and environmental pollution than secondary industries. As the proportion of the service industry increases, the environmental impact on the overall economy may decrease, providing more investment opportunities and cooperation spaces for green finance. In addition, policy support and market demand orientated, consumer demand for environmentally friendly and sustainable products and services increases, and this change in demand may drive financial institutions to increase their supply of green financial products to meet market demand.

In studying the impact of green finance on economic resilience, we found that for every 1% increase in green finance, economic resilience grows by 4.523%, indicating a positive correlation between green finance and economic resilience. The higher the level of green finance development, the stronger the economic resilience and vice versa, thus verifying H3. Green finance can improve the overall resilience and adaptability of society to environmental change by increasing capital investment in green and low-carbon industries, which usually have long-term environmental and economic benefits. Simultaneously, the promotion of green finance helps reduce environmental risks for businesses and the whole economy, as businesses with low-carbon and environmentally friendly technologies typically face lower compliance and environmental risks, making them more flexible and resilient in the face of environmental policies and market changes. Further, the development of green finance promotes technological innovation, as projects supported by green finance often involve the development and application of innovative technologies, such as clean energy technologies, waste management, and circular economy models, the promotion of which can help improve resource efficiency and reduce reliance on traditional polluters, thereby increasing the overall resilience of economic systems.

Table 11 shows the three spatial spillover effects of the model, with the development of the digital economy having a direct effect of 8.219, a total effect of 5.543 and the indirect effct of -2.676 on economic resilience. The direct effect of 8.219 indicates that improvements in the digital economy within a region significantly enhance its economic resilience, likely through increased innovation, productivity, and economic diversification. However, the indirect effect of -2.676 reveals a negative spillover to neighboring regions, suggesting that the growth of the digital economy in one area may come at the expense of others. This could result from resource competition, where regions with a stronger digital economy attract talent, investment, or technological resources away from neighboring areas, thereby weakening their resilience. The total effect of 5.543 reflects the combined impact, which remains positive but smaller than the direct effect alone, highlighting that while the digital economy boosts resilience within regions, its benefits do not spill evenly across space. These findings underscore the importance of coordinated regional policies for mitigating negative spillovers and ensuring balanced economic resilience across regions. From Model (2), the direct effect of digital economy development on green finance is 0.337 and the total effect is 0.230, but the indirect utility is not significant. This finding indicates that digital economy development promotes green finance development through a direct effect rather than a regional spillover effect. The results of Model (3) show that the direct effect of digital economy development on economic resilience was 6.654, the total effect was 5.532, and the indirect effect was not significant. The direct effect of green finance on high-quality economic development is 4.544, but the indirect effect and total effect are not significant, indicating that at the 1% significance level, green finance promotes economic resilience through direct effects rather than regional spillover effects.

Table 12 shows that green finance is positively correlated with corporate innovation and R&D, meaning that for every 1% increase in green finance, corporate innovation improved by 0.917%. This relationship suggests that as investments in green finance grow, companies become more motivated or better equipped to engage in innovative activities and R&D. Innovation and R&D play crucial roles in enhancing economic resilience by enabling businesses to develop new products, adopt sustainable practices, and improve operational efficiency. This leads to increased adaptability and competitiveness in the face of economic shocks and environmental challenges. Thus, green finance indirectly strengthens economic resilience by fostering innovative, sustainable, and shock-resistant economies. Therefore, our findings support H3b, which states that green finance enhances economic resilience by increasing corporate innovation.

**Table 12:** Decomposition of spatial spillover effects.

	Model 1			Model 2			Model 3		
variable	Direct effect	indirect effect	total effect	Direct effect	indirect effect	total effect	Direct effect	indirect effect	total effect
greenf							4.544***	-2.325	2.219
Digf	8.219***	-2.676**	5.543***	0.337***	-0.107	0.230***	6.654***	-1.123	5.532***
ind	0.234***	-1.144***	-0.910***	0.064***	-0.031**	0.033	-0.055	-0.862***	-0.917***
lngdp	0.368***	0.972***	1.341***	0.035***	0.045***	1.080***	0.211***	0.878***	1.089***
popu	0.000**	0.003***	0.003***	0.001***	0.000	0.000***	-0.000*	0.003***	0.003***
open	0.002***	-0.000	0.003**	0.000**	0.000**	0.000***	0.002***	-0.000	0.002*
std	-0.008***	-0.012***	-0.020***	-0.001***	-0.001**	-0.001***	-0.005***	-0.011***	-0.016***
disper	-0.017**	-0.068***	-0.086***	-0.003***	-0.010***	-0.013***	-0.008	-0.035	-0.043*

Green finance is negatively correlated with environmental pollution, meaning that for every 1% increase in green finance, environmental pollution decreases by 8.388%. This reduction in pollution improves public health, preserves ecosystems, and mitigates the risks related to environmental crises. Consequently, green finance enhances economic resilience by promoting a cleaner and more sustainable economy better equipped to withstand and recover from shocks. Therefore, our findings support H3b, which states that green finance enhances economic resilience by reducing environmental pollution.

Compared to Xiao et al. (2024) [61], we investigate the relationship between digital economic development and economic resilience across provinces in China during a similar timeframe. Both studies conclude that the development of the digital economy effectively enhanced China’s economic resilience. However, our study distinguishes itself by introducing green finance as a mediating variable, exploring how the digital economy not only directly impacts economic resilience, but also indirectly through the promotion of green finance. In contrast, Xiao et al. (2024) [61] does not consider green finance; instead, it focuses on the coordinated development and interdependence between the digital economy and economic resilience systems using methodologies such as entropy weight-TOPSIS, coupling theory, spatial autocorrelation, and geodetector analyses.

Comparing with Gu et al. (2024) [62], we examined the factors influencing economic resilience in the context of China’s evolving economy. While Gu also analyze the role of the digital economy, it may focus on different aspects, such as infrastructure development, technological innovation, or policy impacts, without incorporating financial mechanisms, such as green finance. Our study expands this by integrating green finance into the analysis, highlighting its role as a mediator between digital economic development and economic resilience. This approach provides a more nuanced understanding of the pathways through which the digital economy can bolster economic resilience, emphasizing the importance of sustainable financial practices alongside technological advancement.

Our study identifies green finance as a crucial mediating factor in the relationship between digital economic development and economic resilience(Table 13). By demonstrating that the digital economy enhances economic resilience, both directly and indirectly, through the promotion of green finance, our study offers valuable insights into the synergistic effects of technological and financial innovation. This finding underscores the importance of integrating digital economy policies with green finance initiatives to strengthen economic resilience sustainably.

**Table 13:** Results of green finance and economic resiliencies.

	Model (R&D)		Model (Pollution)	
	coefficient	p-value	coefficient	p-value
greenf	0.917**	0.030	-8.388***	0.000
ind	-0.631***	0.000	-0.720***	0.000
lngdp	1.206***	0.000	0.444***	0.000
popdensity	0.0009***	0.031	0.001***	0.000
open	0.002***	0.000	0.001**	0.035
std	-0.002***	0.002	-0.002***	0.182
disper	-0.0206***	0.008	-0.069***	0.000
R^2^	0.9393		0.6459	

### 4.5 Robustness test

To ensure the robustness of our findings, we conducted a robustness test using a latitude- and longitude-based spatial geographic distance matrix instead of a spatial economic geographic weighting matrix, as shown in Table 14. At the 1% level of significance, digital economic development has a significantly positive impact on economic resilience. Similarly, digital economic development has a significant and positive impact on green finance. Additionally, green finance has a significant positive correlation with economic resilience, consistent with the results of the spatial economic geography weight matrix as a weight matrix, indicating that our spatial econometric analyses are robust.

**Table 14:** Spatial econometric results based on the geographical distance weight matrix.

	Model 1		Model 2		Model 3	
	coefficient	p-value	coefficient	p-value	coefficient	p-value
greenf					6.383***	0.000
D	10.351***	0.000	0.4004***	0.000	7.794***	0.000
ind	0.228***	0.000	0.0699***	0.000	-0.218***	0.003
lngdp	0.568***	0.000	0.0366***	0.000	0.334***	0.000
popdensity	0.0006***	0.000	0.0001***	0.000	-0.000	0.317
open	0.0023***	0.702	0.000*	0.124	0.002***	0.000
std	-0.0120***	0.000	-0.0005***	0.026	-0.009***	0.000
disper	-0.0204***	0.001	-0.003***		-0.001	0.826
R^2^	0.8708		0.9150		0.8922	

## 5. Findings and policy recommendations

This study investigates the correlation between digital economic development, green finance, and economic resilience in 30 provinces and cities in China from 2011–2023. Our results show that, first, the development of the digital economy effectively enhances China’s economic resilience. Second, development of the digital economy has effectively promoted green finance. Third, green financing mediated the relationship between digital economic development and resilience. Based on these findings, we make the following recommendations.

Policymakers should prioritize strengthening the digital economy to enhance economic resilience across China’s provinces and cities. This involves investing in digital infrastructure, such as high-speed Internet and next-generation technologies, to ensure widespread connectivity. Additionally, implementing nationwide programs to improve digital literacy and support digital innovation and entrepreneurship can equip the workforce with the necessary skills and foster a culture of innovation.

Promoting green finance is equally crucial as it mediates the positive impact of the digital economy on economic resilience. Policies should focus on expanding green financial instruments such as green bonds and loans, providing financial incentives for sustainable projects, and developing standardized criteria for green investments to ensure transparency. Leveraging financial technology can make green finance more accessible and efficient, for example, by using blockchain for the transparent tracking of green investments.

Finally, an integrated policy approach that combines digital economic development with green finance initiatives maximizes their synergistic effects. This includes facilitating collaboration between technological companies and financial institutions to develop innovative solutions, supporting research and development in green technologies, and strengthening environmental regulations and standards. By adopting these measures, China can achieve sustainable and resilient economic growth that is inclusive, innovative, and environmentally friendly.

## Supporting information

S1 Data (RAR)
